# Supplementary material for: Multi-ancestry GWAS of severe pregnancy nausea and vomiting identifies risk loci associated with appetite, insulin signaling, and brain plasticity
Source: Res Sq. 2024 Dec 16:rs.3.rs-5487737. Preprint. [Version 1] doi: 10.21203/rs.3.rs-5487737/v1 (PMC11702859; doi:10.21203/rs.3.rs-5487737/v1)
Supplement: Supplement 1 [file NIHPPRS5487737v1-supplement-1.pdf]

**Supplementary Figures**

**Supplementary Figure S1.** Locus zoom plots of 10 regions within 1 Mb distance from the 9 lead SNPs in meta-analysis with minimum absolute correlation of 0.3 and coverage of 0.90. One region failed to pass a relaxed genome-wide significant threshold ($P < 5 \times 10^{-6}$) was removed. **A.** chr3:118.0-119.0 Mb **B.** chr4: 57.0-58.0 Mb **C.** chr6:54.8-55.8 Mb **D.** chr10:112.8-113.8 Mb **E.** chr11:29.6-34.6 Mb **F.** chr11:100.8-110.8 Mb **G.** chr13:83.5-84.5 Mb **H.** chr19:17.9-18.9 Mb **I.** chr22:32.5-33.5 Mb

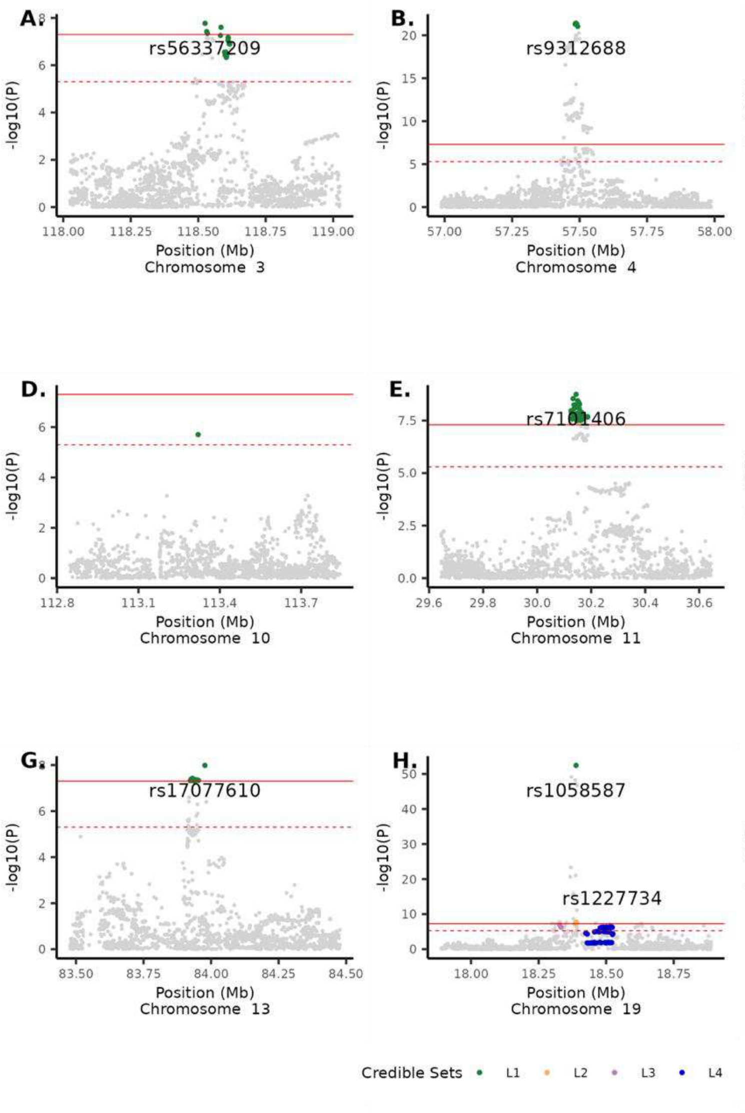

**Supplementary Figure S2.** Regional association plots for 10 SNPs found in the meta-analysis.

**A.** rs56337209 **B.** rs9312688 **C.** rs10073299 **D.** rs10948901 **E.** rs76856932 **F.** rs7101406 **G.** rs12790159 **H.** rs17077610 **I.** rs1058587 **J.** rs5994661

**A.** rs56337209

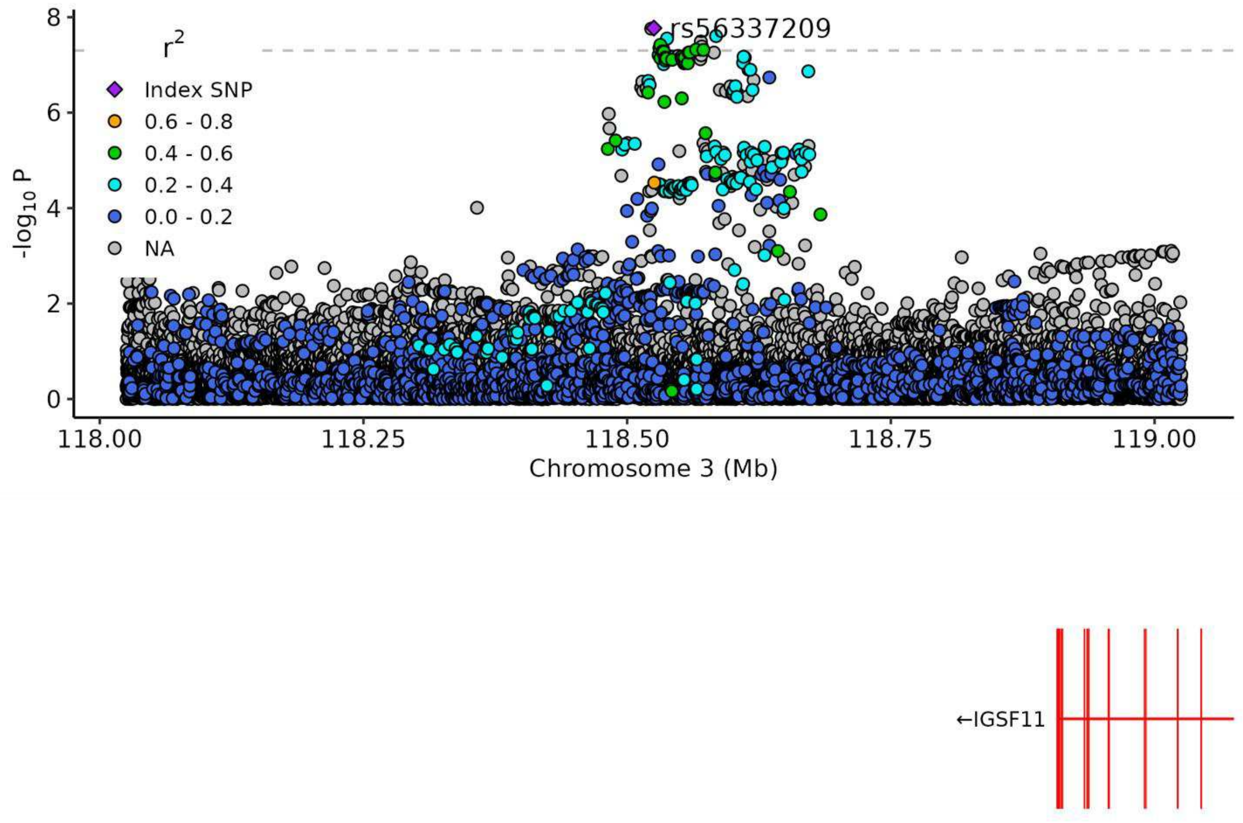

**B.** rs9312688

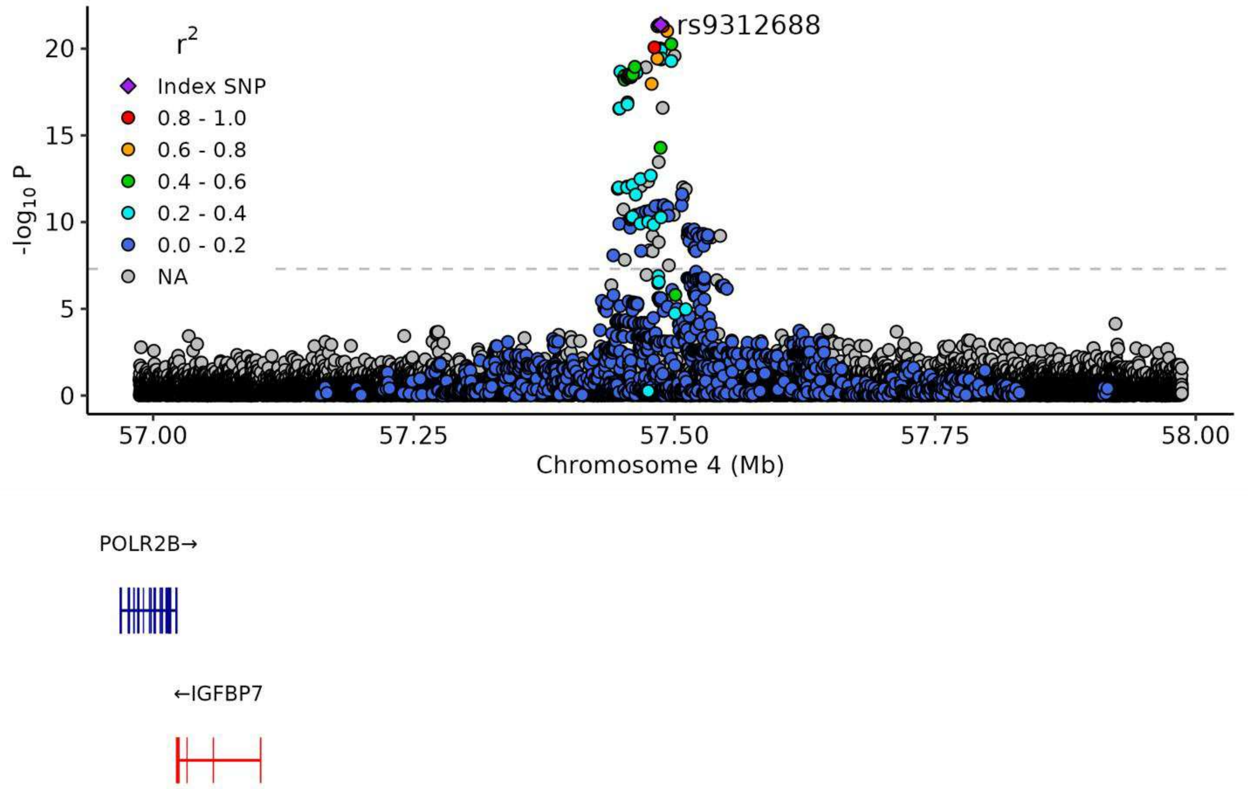

rs9312688
r2
Index SNP
0.8 - 1.0
0.6 - 0.8
0.4 - 0.6
0.2 - 0.4
0.0 - 0.2
NA
-log10 P
0
5
10
15
20
57.00
57.25
57.50
57.75
58.00
Chromosome 4 (Mb)
POLR2B→
←IGFBP7

**C.** rs10073299

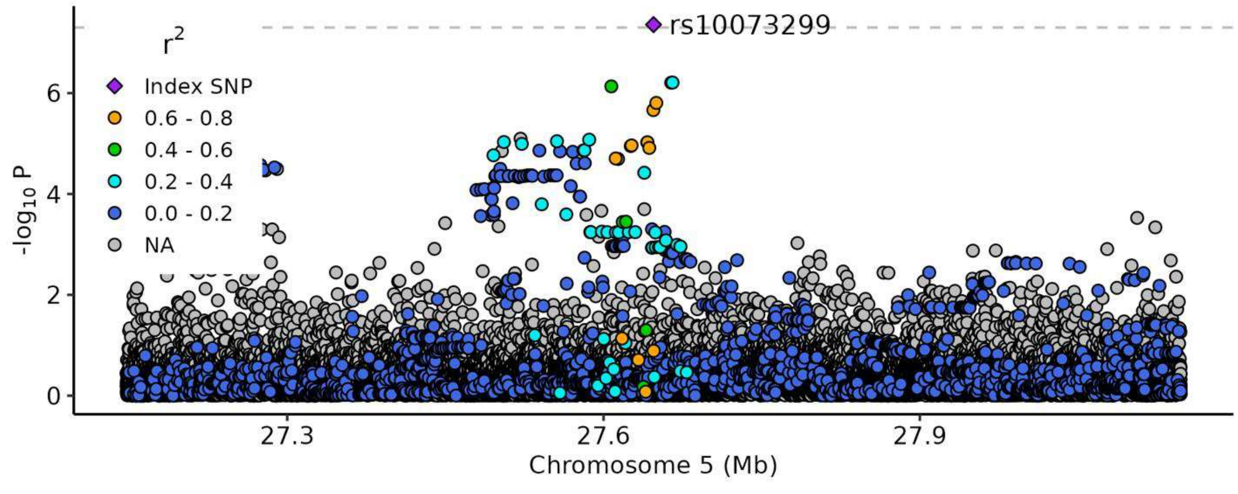

**D.** rs10948901

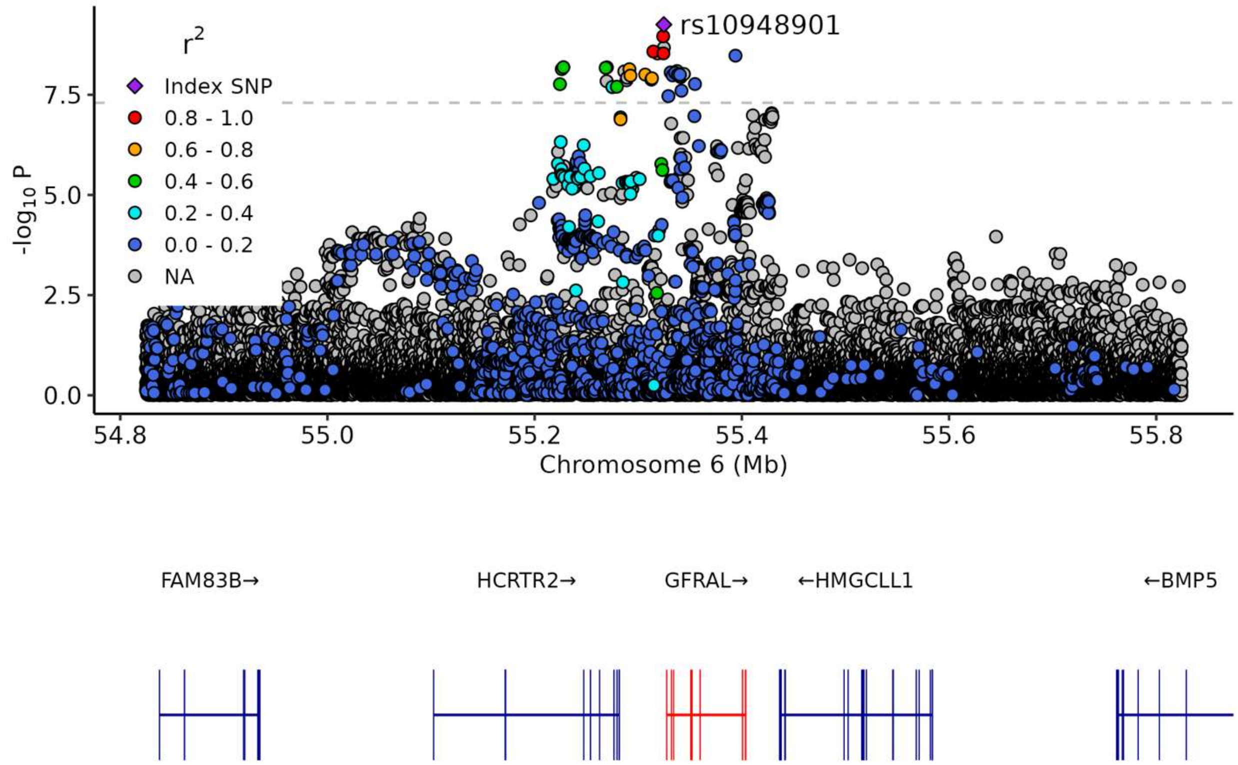

**E.** rs76856932

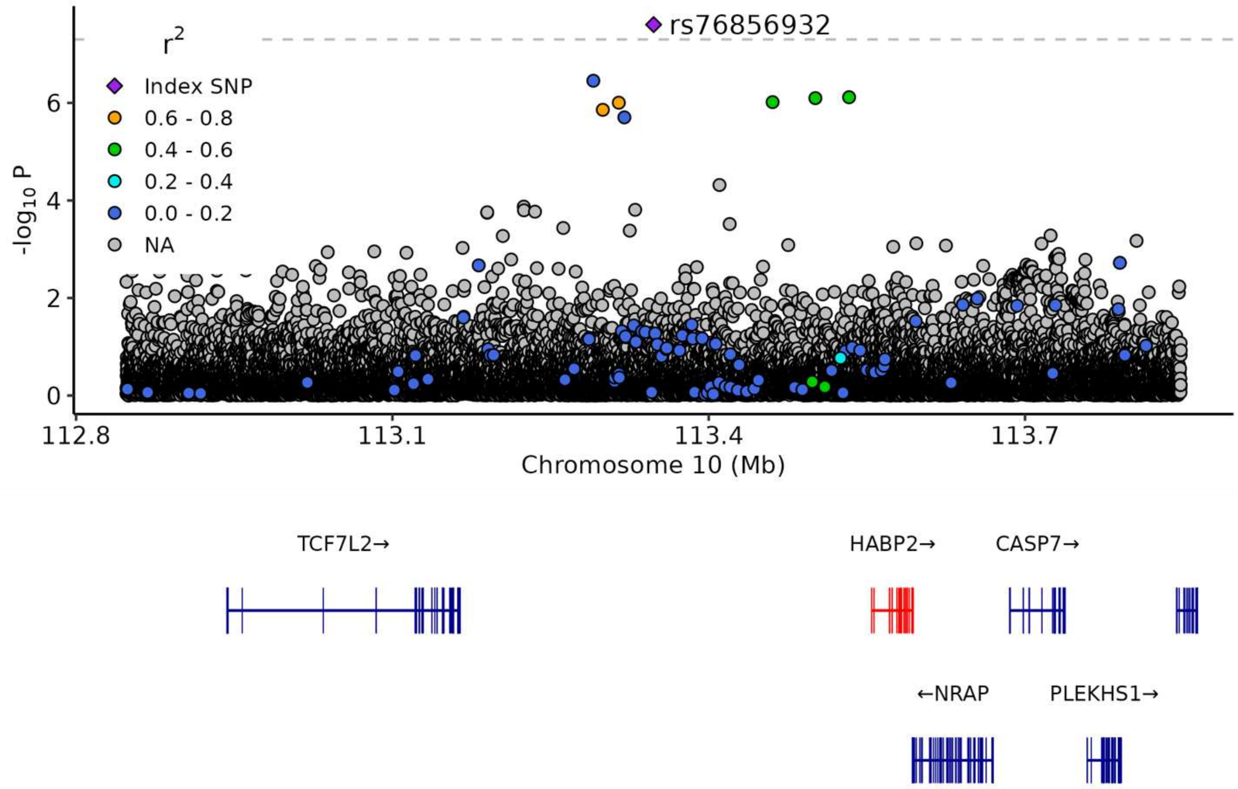

**F.** rs7101406

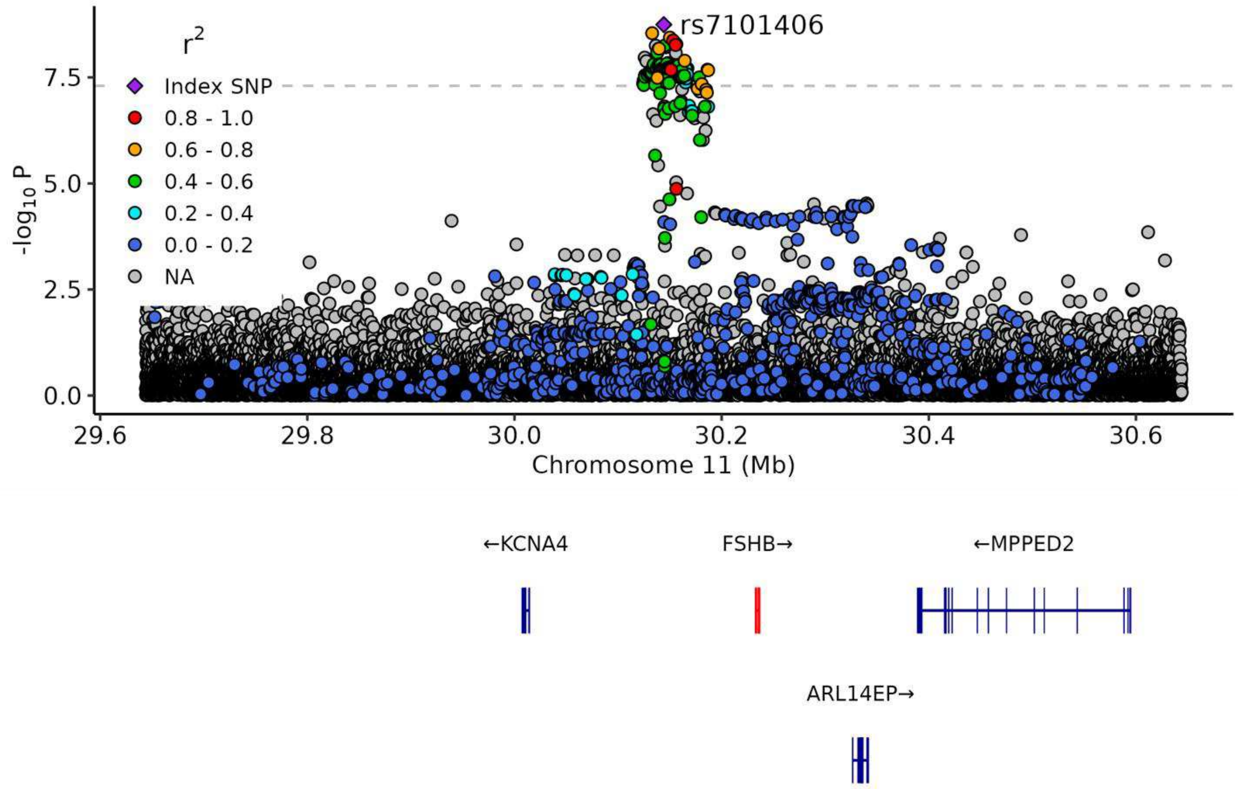

rs7101406
r²
Index SNP
0.8 - 1.0
0.6 - 0.8
0.4 - 0.6
0.2 - 0.4
0.0 - 0.2
NA
-log₁₀ P
7.5
5.0
2.5
0.0
29.6
29.8
30.0
30.2
30.4
30.6
Chromosome 11 (Mb)
←KCNA4
FSHB→
←MPPED2
ARL14EP→

**G.** rs12790159

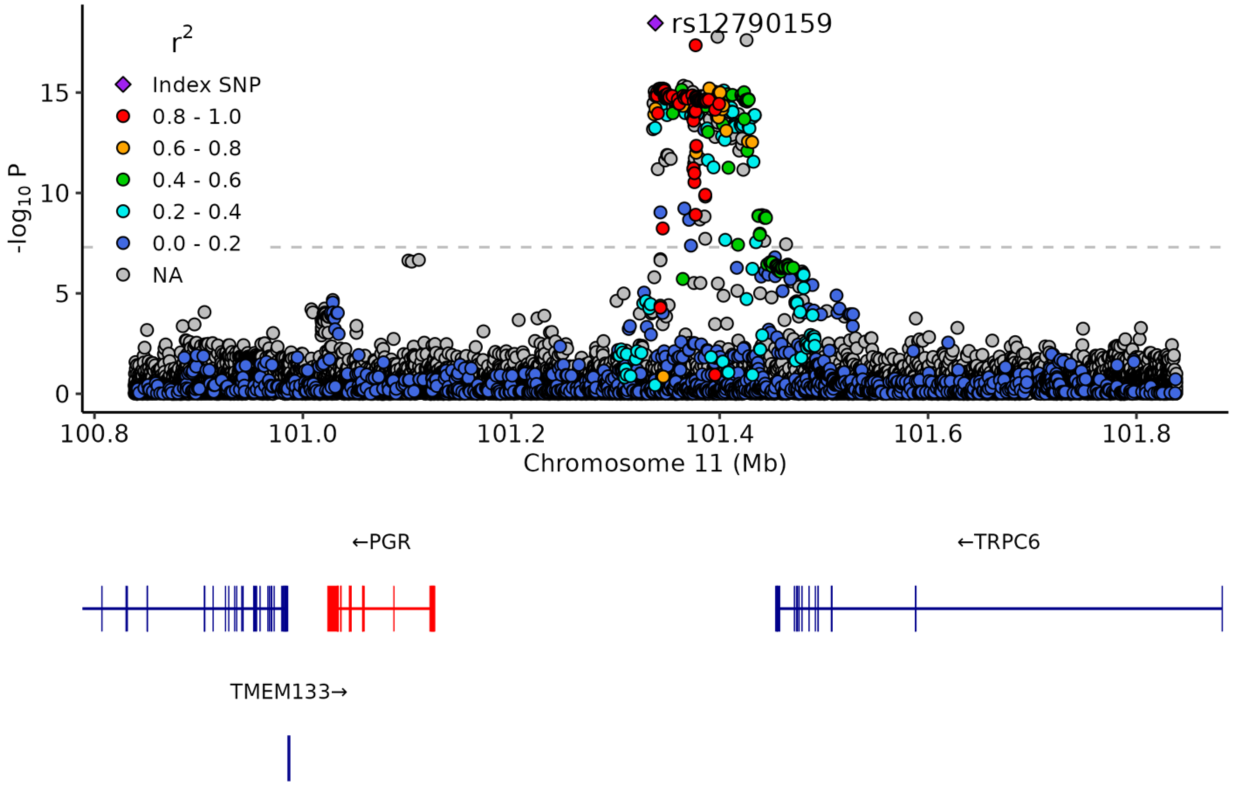

**H.** rs17077610

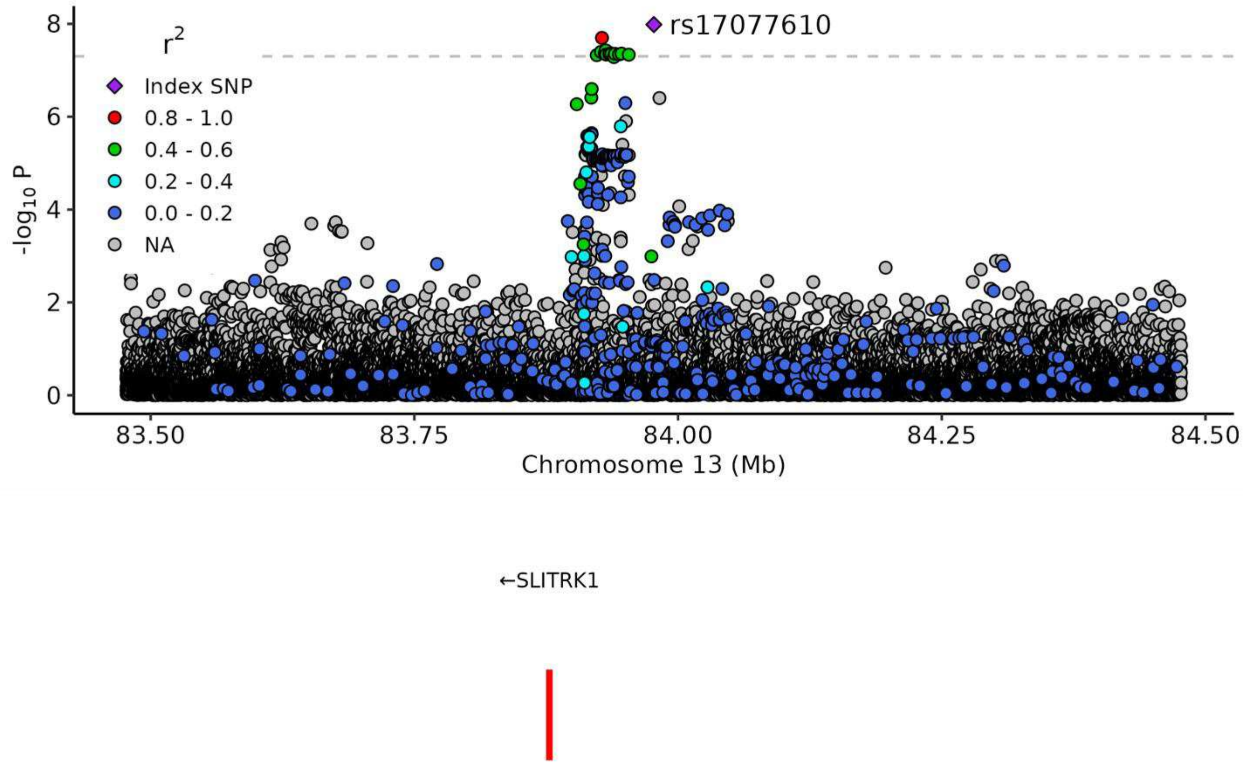

**I.** rs1058587

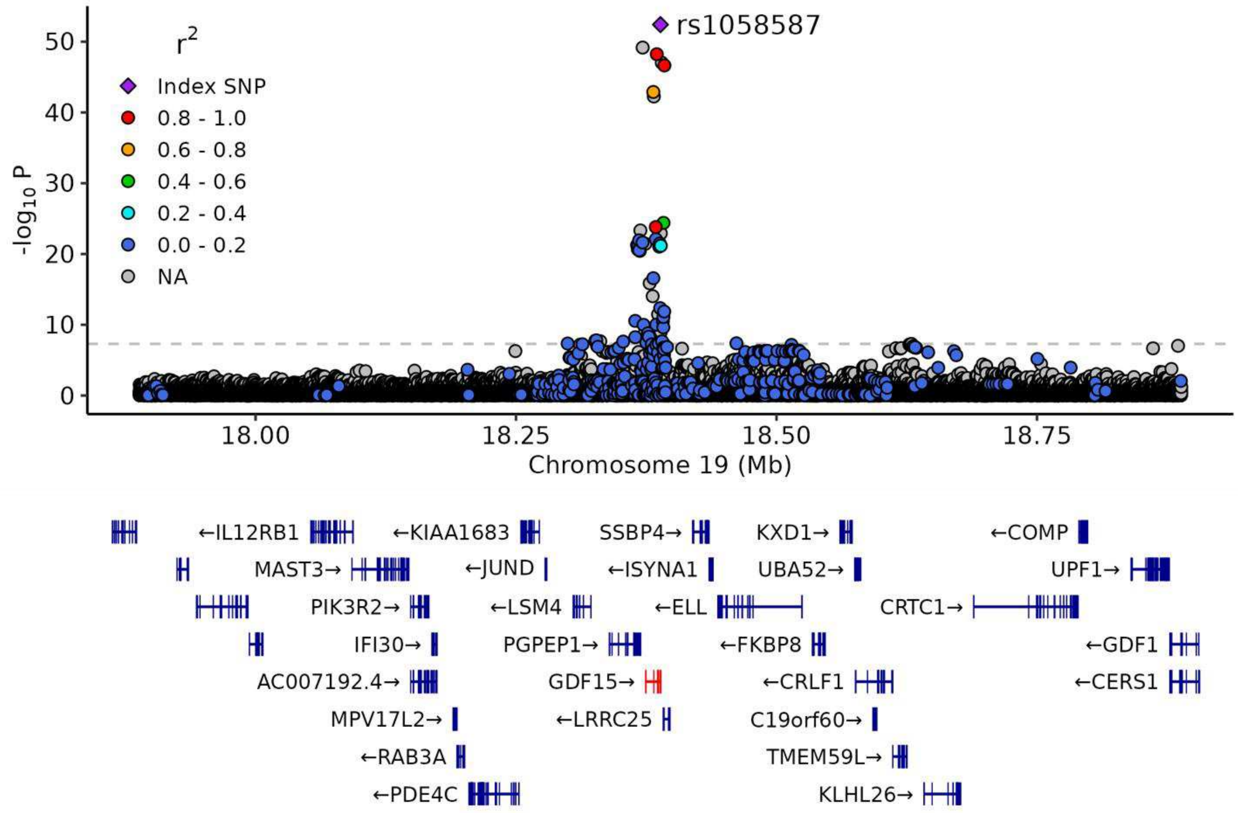

rs1058587
r²
Index SNP
0.8 - 1.0
0.6 - 0.8
0.4 - 0.6
0.2 - 0.4
0.0 - 0.2
NA
-log₁₀ P
50
40
30
20
10
0
18.00
18.25
18.50
18.75
Chromosome 19 (Mb)
←IL12RB1
←KIAA1683
SSBP4→
KXD1→
←COMP
MAST3→
←JUND
←ISYNA1
UBA52→
UPF1→
PIK3R2→
←LSM4
←ELL
CRTC1→
IFI30→
PGPEP1→
←FKBP8
←GDF1
AC007192.4→
GDF15→
←CRLF1
←CERS1
MPV17L2→
←LRRC25
C19orf60→
←RAB3A
TMEM59L→
←PDE4C
KLHL26→

**J.** rs5994661

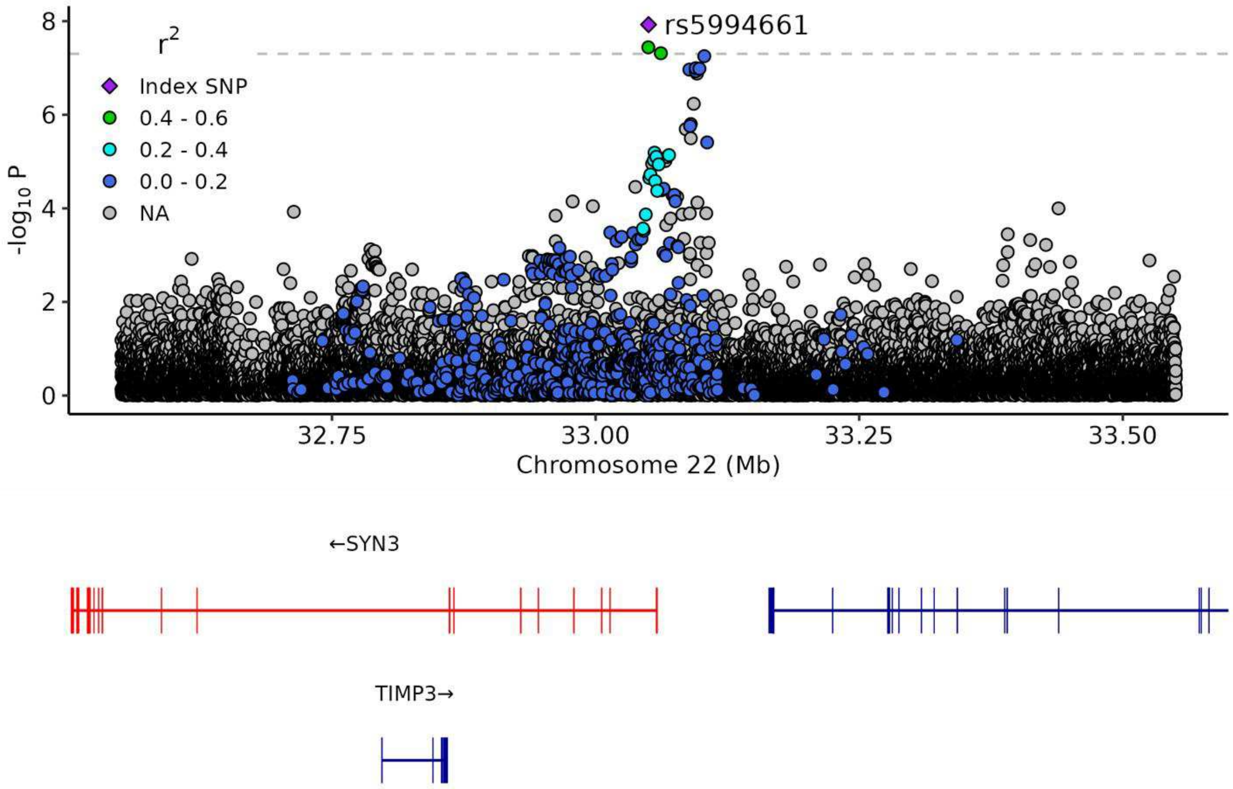
rs5994661
r2
Index SNP
0.4 - 0.6
0.2 - 0.4
0.0 - 0.2
NA
-log10 P
8
6
4
2
0
32.75
33.00
33.25
33.50
Chromosome 22 (Mb)
←SYN3
TIMP3→

**Supplementary Figure S3.** QQ Plot

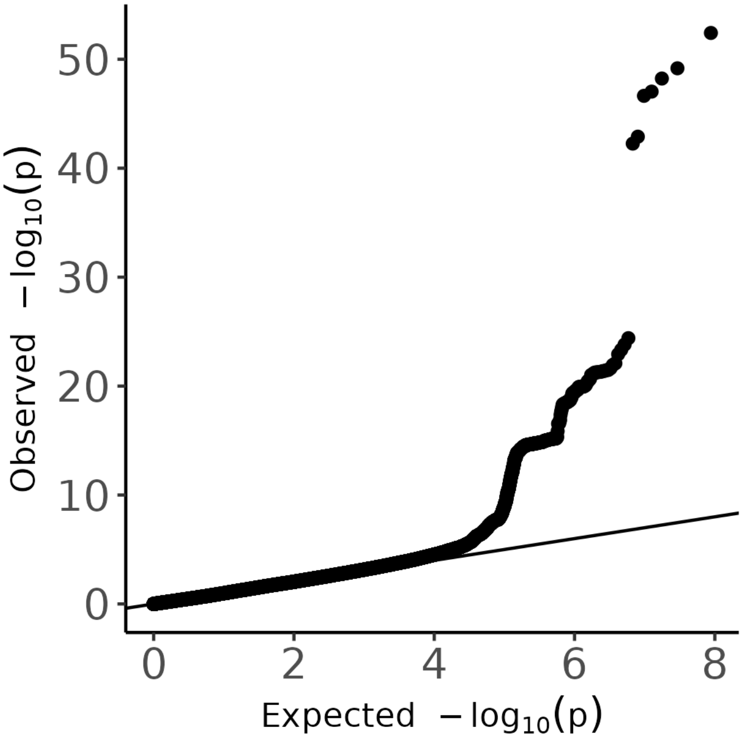

**Supplementary Figure S4**. No significant difference in pro-GDF15 maturation between H (GDF15) and D (H202D) for rs1058587.

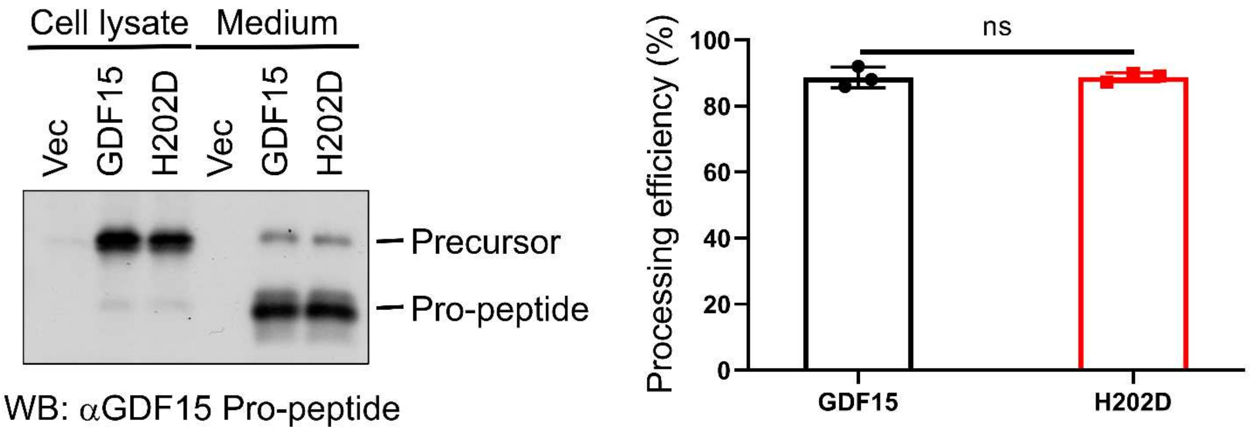

# Supplementary Files

This is a list of supplementary files associated with this preprint. Click to download.

- TABLES13.docx
- SUPPLEMENTARYTABLESS16.docx
